# Fatal Epistaxis

**Published:** 1831-01-01

**Authors:** 


### REGIMENTAL HOSPITAL, 13TH LIGHT INFANTRY.

### FATAL EPISTAXIS.

IN the fifth volume of the Calcutta Transactions, just received, we find the following report by Surgeon Seevewright.

*Case.* Corporal Ambler, aged 40, was admitted into hospital on the 28th September, 1826, in a state of profuse hæmorrhage from the nose, confined chiefly to the right nostril. It had commenced two hours previously, in his barrack-room, and it appeared that he had lost a considerable quantity of blood before he was brought into the hospital. He had been inebriated for several days, and, on the 26th September, had fallen down a flight of stairs and injured his head and nose. Wet cloths were applied to the head, and an attempt was made to plug the nostrils. A sense of suffocation prevented the execution of this measure. A dossil of lint, dipped in spirits, was introduced into the nostril, and, for a short time, checked the hæmorrhage. Afterwards a strong solution of the cupri sulphas was used, but produced sickness. The hæmorrhage ceased and returned through the day. The patient passed a quiet night; the hæmorrhage came on fresh on the morning of the 29th, and next day he expired.

On dissection, it was observed that the dura mater adhered with exceeding little tenacity to the skull. The vessels of the brain, at first sight, appeared congested; but, on more minute examination, many of them were found empty, and others containing a colourless fluid. The serous effusion between the membranes and in the ventricles, amounted to upwards of six ounces. The substance of the brain itself was firm. A large coagulum of blood was traced from the left nostril, communicating with the frontal sinus. There was a similar one on the other side. The right antrum maxillare was filled with a clot of blood. The precise source of the hæmorrhage does not appear to have been ascertained.

